# SFTA3 – a novel surfactant protein of the ocular surface and its role in corneal wound healing and tear film surface tension

**DOI:** 10.1038/s41598-018-28005-9

**Published:** 2018-06-28

**Authors:** Martin Schicht, Fabian Garreis, Nadine Hartjen, Stephanie Beileke, Christina Jacobi, Afsun Sahin, Detlef Holland, Henrik Schröder, Christian M. Hammer, Friedrich Paulsen, Lars Bräuer

**Affiliations:** 1 Department of Functional and Clinical Anatomy, Friedrich-Alexander-University Erlangen-Nürnberg (FAU), Erlangen, Germany; 2 Department of Ophthalmology, Friedrich-Alexander-University Erlangen-Nürnberg (FAU), Erlangen, Germany; 3 Ophthalmological Practice Tibarg, Hamburg, Germany; 4 Department of Ophthalmology, Eskisehir Osmangazi University Hospital, Eskisehir, Turkey; 5 Eye Clinic Bellevue, Kiel, Germany

## Abstract

The study aimed to characterize the expression and function of SFTA3 at the ocular surface and in tears. Ocular tissues, conjunctival (HCjE) and human corneal (HCE) epithelial cell lines as well as tearfilm of patients suffering from different forms of dry eye disease (DED) were analyzed by means of RT-PCR, western blot, immunohistochemistry, and ELISA. A possible role of recombinant SFTA3 in corneal wound healing was investigated performing *in vitro* scratch assays. Tear film regulatory properties were analyzed with the spinning drop method and the regulation of SFTA3 transcripts was studied in HCE and HCjE after incubation with proinflammatory cytokines as well as typical ocular pathogens by real-time RT-PCR and ELISA. The results reveal that human ocular tissue as well as tears of healthy volunteers express SFTA3 whereas tears from patients with DED showed significantly increased SFTA3 levels. *In vitro* wounding of HCE cell cultures that had been treated with recombinant SFTA3 demonstrated a significantly increased wound closure rate and rSFTA3 reduced the surface tension of tear fluid. The results indicate that SFTA3 at the ocular surface seemed to be involved in wound healing and the reduction of surface tension.

## Introduction

In Central Europe, about 15% to 20% of the human population are suffering from dry eye disease (DED, keratoconjunctivitis sicca). Exogenous influences (e.g. contact lenses, air, drugs, diet) and endogenous factors (e.g. age, hormonal imbalances, diabetes mellitus) are the main causes of this disease. DED is based upon a discontinuity of the tear film^1^ (DEWS Report 2007) characterized by cardinal symptoms like burning, itchy and watery eyes, painful eye pressure, oppressive feeling, foreign body sensations and light sensitivity. In almost 86% DED is the result of a diminished outer lipid layer at the surface of the tear film^2^, a condition called evaporative DED. A quantitative deficit of tears, on the other hand, is considered to be less common. The lipid component of the tear film is mainly secreted by the meibomian glands of the eyelids. Under physiological conditions, the lipid component prevents evaporation and early break-up of the tear film and thus, protects the eye surface^3^. Together with the lacrimal glands and accessory lacrimal glands of the eyelids which produce the aqueous component of the tear film, meibomian secretions (meibum) generate a barrier against pathogenic microorganisms^4^.

A further component of the tear film comprises proteins of the surfactant family (SP-A, -B, -C and –D) that are well known from the lung and have previously also been described at the eye surface, in tear film and in the lacrimal gland^5–7^. In the lung surfactant proteins regulate surface activity and have immunological properties with regard to both adaptive and innate immunity^8–10^.

Recently, two novel surfactant proteins SFTA2 (SP-G)^11^ and SFTA3 (SP-H)^12^ were identified and characterized. SP-G has physicochemical properties similar to the already described proteins SP-B and SP-C. Moreover, the ability to interact with lipid systems combined with a potential surface-regulatory function of SP-G has been discussed. Surfactant-associated protein 3 (SFTA 3) was first identified by bioinformatic analyses and named surfactant protein H (SP-H) or SFTA3^13^. This protein shows no significant sequential or structural similarities with other surfactant proteins or other known proteins in general. The proposed physicochemical properties of SFTA3 show similarities with the well-characterized surfactant proteins B and C. Molecular dynamic (MD) simulations and protein modeling analyses suggest that SFTA3 may constitute a surface-active substance. Despite the absence of known structurally conserved immune regulatory domains SFTA3 might still play a possible role in the immune defense and during inflammation processes^12^. Stimulation experiments with a lung epithelial cell line (A549) revealed that SFTA3 gene expression is significantly influenced by the presence of immunomodulating cytokines (IL-1β and IL-23) as well as bacterial lipopolysaccharide (LPS)^12^. In a dose-effect analysis with IL-1β and IL-23 a reduced expression of SFTA3 was shown, while LPS induced SFTA3 expression. It was hypothesized that LPS simultaneously triggers the production of inflammatory cytokines like IL-1β and IL-23 production in resident alveolar macrophages and SFTA3 induction via TLR in alveolar epithelial cells. Thus, gram-negative bacterial infection apparently promotes the SFTA3 pathway in alveolar epithelial cells, whereas inflammatory cytokines like IL-1β and IL-23 secreted by alveolar macrophages apparently limit this process^12^. A recent study on the impact of recombinant SFTA3 protein on alveolar macrophages showed an increased phagocytosis rate, suggesting a possible immunological function of the protein inside the lung^14^.

In the present study we investigated whether SFTA3 is also part of the tissues of the human ocular surface, the lacrimal system as well as tears. Furthermore, we examined the expression and secretion of SFTA3 (SP-H) at the human ocular surface and in tear fluid samples of patients with dry eye disease (DED).

## Material and Methods

### Study design

The objective of this study was to determine the expression and production of SFTA3 at the healthy human ocular surface and to investigate potential differences in the presence of dry eye disease (DED). SFTA3 expression was analyzed by western blot analysis, ELISA as well as by immunohistochemistry and immunofluorescence in different tissues of the ocular surface and in human tear fluid from patients suffering from DED in comparison to healthy controls. The activation and regulation of SFTA3 transcription was studied in cell culture experiments after incubation with ocular pathogens and cytokines by real-time RT-PCR and ELISA. A possible contribution of SFTA3 in corneal wound healing was investigated by an *in vitro* scratch assay and physicochemical tear film regulatory properties was analyzed with the spinning drop method.

### Sample generation

#### Tissues

The preparation of the samples were performed as previously described by Schicht *et al*.^15^. All tissue samples were obtained from body donors (5 male, 11 female, aged 33–76 years) donated to the Department of Anatomy and Cell Biology, Martin Luther University Halle-Wittenberg, Germany. All body donors signed a testamentary permission in time. The study included proper consent and approval, complied with the tenets of the Declaration of Helsinki, and was approved by institutional review board regulations. The samples were obtained from the body donors within a time-frame of 5–20 h post mortem. Prior to dissection, the history of each cadaver was studied. The donors were free of recent trauma, eye and nasal infections or diseases involving or affecting lacrimal function. Moreover, with regard to the whole body, donors that had been affected by acute infection, tumors, recent trauma or surgical operations were not used in this study. After the dissection of cornea, conjunctiva, lacrimal gland, eyelid and efferent tear ducts (lacrimal sac and nasolacrimal duct), one half of each specimen was fixed in 4% paraformaldehyde for later paraffin embedding. The other half was used for molecular-biological investigations and was thus immediately frozen at −80 °C. Bronchial mucosa and lung samples served as positive control tissues.

#### Tear fluid

Tear fluid extraction were performed as previously described by Schicht *et al*.^15^. Tear fluid samples were collected with Schirmer strips at the Department of Ophthalmology, Friedrich Alexander University Erlangen-Nürnberg, Germany. These specimens were obtained in compliance with good clinical practice and with informed consent (54-2532.1-35/13). Ethical approval was obtained from the Ethics Committee of the University of Erlangen-Nürnberg. Written consent was received from all patients and subjects after explanation of the procedures and study requirements. Tears from 21 control subjects (mean age 42.8 ± 8.3 years; 9 women and 13 men) were included in the study. They showed no symptoms for dry eye disease or ocular discomfort, did not use any artificial tears or lubricant eye drops and did not suffer from any autoimmune disorders or other eye diseases including ocular allergies and had no history of eye surgery or contact lens wearing. 39 eyes of 39 patients (mean age 42.8 ± 8.3 years; 20 women and 19 men) with moderate to severe dry eye (DEWS dry eye severity level 2)^16^ were also enrolled in the study. Inclusion criteria were as follows: (i) Ocular Surface Disease Index questionnaire Score (OSDI Score) >40, (ii) tear break-up time (TBUT) ≤10 s, (iii) Schirmer test with anesthesia ≤10 mm, (iv) lid-parallel conjunctival folds (LIPCOF) >2. A more detailed description of these points is given below. For the ELISA, patients were divided into two subgroups: evaporative dry eye (EDE, n = 14) and aqueous-deficient dry eye (ADDE, n = 14). EDE patients had a tear break-up time of ≤5 s and a Schirmer test ≤10 mm. ADDE patients showed a tear break-up time of ≤10 s and a Schirmer test ≤5 mm. Exclusion criteria consisted of a medical history of trauma or infection, ocular allergies, pregnancy, lactation, history of refractive surgery/ocular surgery/any other surgery within the previous 6 months, immunosuppressive medications, or the use of contact lenses within 14 days prior to ophthalmological examination. Moreover, patients wearing punctum plugs, patients with history or evidence of epithelial keratitis derived from herpes simplex infection, recent varicella infection, corneal or conjunctival viral disease, acute corneal, conjunctival, or palpebral bacterial infection; or ocular fungal infection were excluded from this study.

### Cell culture

Cell culture experiments were performed as previously described by Schicht *et al*.^15^. SV40-transformed human corneal epithelial cells (HCE cells, obtained from Kaoru Araki-Sasaki, Tane Memorial Eye Hospital, Osaka, Japan, passage number 18–27)^17^ as well as a human spontaneously immortalized epithelial cell line from normal human conjunctiva [IOBA-NHC, here referred to as HCjE cells, obtained from Yolanda Diebold, University Institute of Applied Ophthalmobiology (IOBA), University of Valladolid, Valladolid, Spain]^18^ were cultured as monolayer and used for further stimulation experiments. HCE and HCjE cells were cultured with Dulbecco’s Modified Eagle Medium (DMEM)/HAM’s F12 (1:1) (PAA Laboratories GmbH, Pasching, Austria) supplemented with 10% fetal calf serum (FCS, Biochrom AG, Berlin, Germany) in a humidified incubator containing 5% CO_2_ at 37 °C. For stimulation experiments, cells (1 × 10^6^) were seeded in Petri dishes and cultured until confluence was reached. Cells were washed with phosphate buffered saline (PBS) and changed to serum-free medium for 3 h. Afterwards, cells were either treated with recombinant proinflammatory cytokine interleukin (IL)-1β (10 ng/ml, ImmunoTools, Friesoythe, Germany) or tumor necrosis factor (TNF) α (10 ng/ml, ImmunoTools, Friesoythe, Germany) for 6 h, 12 h, 24 h, or 48 h each, or with different dilutions of bacterial supernatants of *Staphylococcus aureus* (SA) or *Pseudomonas aeruginosa* (PA) for 6 h, 24 h or 48 h. Tryptone Soy Broth (TSB) was used to incubate the bacteria. Therefore, incubation of cells with TSB that had not been exposed to bacteria served as a medium control in the bacterial supernatant group. In the cytokine group incubation with the cytokine solvent alone yielded the controls. All experimental procedures were performed under normoxic conditions. On completion of each experiment, cells and culture supernatants were collected and stored at −80 °C until they were processed for RNA extraction (cells) or analysis of SFTA3 secretion (culture supernatants). For *in vitro* simulation of wound healing, a scratch experiment was conducted using confluent HCE cells. The confluent monolayer was scratched three times with a sterile 100 μl pipette tip, washed three times with PBS and incubated with serum-free cell culture media for different time spans (0, 15, 30 min and 1, 3, 6 h). All experimental procedures were performed under normoxic conditions. On completion of each experiment, cells were harvested by using the peqGOLD TriFast System and stored at −80 °C until they were processed for RNA extraction.

### Production of bacterial supernatants

Extraction of bacterial supernatants was performed as previously described by Schicht *et al*.^15^. Laboratory strains of *S*. *aureus* and *P*. *aeruginosa* were grown overnight at 37 °C by shaking in Tryptone Soy Broth (Oxoid, Basingstoke, England). Bacteria were centrifuged (Scanspeed 1730R) twice at 6,000 rpm/3393 rcf for 30 min. Supernatants were filtered twice using filters impermeable to bacteria (0.22 µm pore size; Millipore, Eschborn, Germany). Aliquots of the supernatants were proven to be sterile by overnight incubation on agar. TSB growth medium (diluted 1:100) served as medium control.

### Patient examination

Patient examination after OSDI Score, LOPCOF and TBUT was performed as previously described by Jacobi *et al*.^19^ and Schicht *et al*.^15^.

#### OSDI Score questionnaire

The OSDI is a subjective symptom questionnaire that measures the severity of dry eye disease. It includes 12 items regarding visual function, ocular symptoms, and environmental triggers queried for the past week. The questionnaire is standardized and well described by Schiffmann *et al*.^20^. The OSDI was already successfully used to measure the outcome in a randomized controlled trial^21^.

#### LIPCOF (Degree 0–3)

LIPCOF were evaluated by slit-lamp examination. The classification of LIPCOF according to Höh *et al*. was used^22^. Here, degree 0 means that no permanent conjunctival fold exist. Degree 1 describes the permanent presence of one single fold which does not exceed the height of the normal tear meniscus. With degree 2, the LIPCOF disintegrates into 2 or several small parallel folds that remain lower than the normal tear meniscus. If there are several parallel conjunctival folds exceeding the height of the normal tear meniscus, degree 3 is present.

#### TBUT (s)

For diagnosing tear film stability, a standardized measurement of the tear film break-up time was taken. 5 μl of non-preserved 2% sodium fluorescein were instilled onto the bulbar conjunctiva (the secretion of reflex tears was prevented by using a micropipette). Then the patient was instructed to blink normally without squeezing several times to distribute the fluorescein and then refrain from blinking until told otherwise. The slit lamp magnification was set at 10× and a Wratten 12 yellow filter was used to enhance the observation of the tear film over the entire cornea^23^. A stopwatch was used to record the time between the last complete blink and the first indication of tear film break-up. Thereafter, the patient was instructed to blink normally again. Three measurements were taken as recommended by the DEWS II report^24^, and the average was calculated.

### Schirmer’s test

Schirmer’s test with anesthesia (eyes closed, mm) was performed as previously described by Schicht *et al*.^15^.

After topical anesthesia with one drop of oxybuprocaine-HCL (*Conjuncain-EDO®*), a Schirmer test strip (35 × 5 mm; Liposic-Schirmer-Test-Streifen, Dr. Mann Pharma, 13581 Berlin, Germany) was placed in the lower outer fornix and then the patient was instructed to close his/her eyes. After 5 minutes, the strip was removed from the eye and the length of the wetted Area was measured^21^. In addition, the tear fluid-soaked Schirmer strip was transferred to a 1.5 ml reaction tube and stored at −80 °C for further experiments. For extraction of the tear fluid, the Schirmer strips were transferred to a 0.5 ml tube punctured at the bottom with a cannula. The tube was placed in a larger (1.5 ml) tube and centrifuged at maximum rpm (17900 rcf) for 5 minutes. The centrifugal force pulled the tear fluid out of the Schirmer strip, through the central “pore” in the bottom of the smaller tube and into the outer 1.5 ml tube^25^.

### Molecularbiological analyses

RNA preparation, cDNA synthesis as well as RT-PCR analysis were performed as previously described by Schicht *et al*.^15^.

#### RNA preparation and cDNA synthesis

For the RT-PCR, tissue biopsies were crushed in an agate mortar under liquid nitrogen and homogenized (Polytron, Norcross, GA). Total RNA was extracted from the samples by use of the RNeasy Mini Kit (Qiagen, Hilden, Germany).

In addition, total RNA was extracted from cultivated HCE and HCjE (PeqGold reagent; PeQLab, Erlangen, Germany). Crude RNA was purified with isopropanol and repeated ethanol precipitation, and contaminated DNA was destroyed by digestion with RNase-free DNase I (30 minutes 37 °C; Boehringer, Mannheim, Germany). The DNase was heat-inactivated for 10 min at 65 °C. Reverse transcription of all RNA samples to first-strand cDNA (RevertAid H Minus M-MuL V Reverse Transcriptase Kit; Fermentas, St. Leon-Rot, Germany) was performed according to the manufacturer’s protocol. Two micrograms total RNA and 10 pmol Oligo (dT)_18_ primer (Fermentas) were used for each reaction. The ubiquitously expressed β-actin and HPRT which proved amplifiable in each case with the specific primer pair, served as the internal control for the integrity of the translated cDNA.

#### Polymerase Chain Reaction (PCR)

Conventional PCR was performed according to the following protocol: 1. 95 °C for 5 min, 2. 95 °C for 30 sec, 3. 62 °C for 30 sec, 4. 72 °C for 30 sec, 5. 72 °C for 5 min. Steps 2. −4. were repeated 30 times. Primers used for conventional PCR: SFTA3 sense 5′-ATG AGA GCC GGG TTT TCT GAC-3′, antisense 5′-TGC AGT ATG AAT AAT TAA CAT C-3′ (282 bp), as well as for quantitative (i.e. Real Time) PCR were: SFTA3 real sense 5′-TGG TGT TCC AAA TAT TGC AGA-3′, antisense 5′-GTT CAT CCG AGG CCA AGA-3′ (92 bp) and 18S human sense 5′-GGT GCA TGG CCG TTC TTA-3′, antisense 5′-TGC CAG AGT CTC GTT CGT TA-3′. PCR products were confirmed by Sanger-sequencing, using the BigDye Terminator v3.1 Cycle Sequencing Kit (Applied Biosystems, Foster City, CA). To estimate the amount of amplified PCR product, we performed a β-actin PCR with specific primers: sense 5′-CAA GAG ATG GCC ACG GCT GCT-3′, antisense 5′-TCC TTC TGC ATC CTG TCG GCA-3′ (275 bp) for each analyzed tissue.

#### Quantitative Real-Time RT-PCR

Gene expression was analyzed with quantitative Real-Time RT-PCR (qPCR) using a LightCyler480^®^ system (Roche). The PCR reaction mixture contained 10 μL LightCycler480^®^ 5x probe mastermix, 0.25 μL of each primer and 2 μL of each cDNA, 0,4 µl Universal ProbeLibrary (UPL, Roche) probe #13 for SFTA3 or #22 for 18S (10 µM) and 7,1 µl nuclease free water. On each 96-well plate qPCR was performed with a cycle of 5 min at 95 °C, 55 cycles of 15 s at 95 °C, 30 s at 60 °C and 1 s at 72 °C, to confirm amplification of specific transcripts. SFTA3 and 18S primers as well as the corresponding UPL probes (see above) were generated by using the ProbeFinder^TM^ software (Version 2.04, Roche). A standard curve was generated by serial dilutions of cDNA from non-stimulated cells. To standardize mRNA concentration the transcript levels of the housekeeping gene small ribosomal subunit (18S rRNA) were determined in parallel for each sample, and relative transcript levels were corrected by normalization based on the 18SrRNA transcript levels. All qPCRs were performed in triplicate, and the changes in gene expression were calculated applying the ΔΔC_t_ method.

### Western blot analysis

Western blot analysis was performed as previously described by Schicht *et al*.^12^.

For Western blot analysis tissue samples (standardized ratio: 100 mg wet weight/400 µm buffer containing 1% SDS and 4% 2-mercaptoethanol) were extracted as previously described^26^. The protein concentration was measured with a protein assay based on the Bradford dye-binding procedure (BioRad, Hercules, CA). The total protein (30 µg) was then analyzed by western blot. Proteins were resolved by reducing 15% SDS-polyacrylamide gel electrophoresis and horizontally transferred at room temperature for 2 h at 0.8 mA/cm^2^ onto a nitrocellulose membrane with a pore size of 0,1 µm. Bands were detected with a polyclonal primary antibody to SFTA3 (1:200) and a secondary antibody (anti-rabbit IgG, respectively, conjugated to horseradish peroxidase, 1:2.000) using chemiluminescence (ECL- Plus; Amersham-Pharmacia, Uppsala, Sweden). To verify the specificity of the self made antibody employed, two additional commercial SFTA3 antibodies were used: goat anti SFTPH – D14 (Santa Cruz, sc-248574) and Goat anti SFTPH – C20 (Santa Cruz, sc-24876). Protein extracted from human lung and bronchoalveolar lavage (BAL) served as positive control. The molecular weights of the detected protein bands were estimated using standard proteins (Prestained Protein Ladder, Fermentas, St. Leon-Rot, Germany) ranging from 10 to 170 kDa.

### Immunohistochemistry

Immunohistochemistry analysis was performed as previously described by Schicht *et al*.^12^.

For immunohistochemistry, specimens taken from healthy body donors tissues were embedded in paraffin, sectioned (5 µm) and dewaxed. Immunohistochemical staining was carried out with a self made polyclonal primary antibody against SFTA3. Antigen retrieval was performed by microwave pretreatment for 10 min and non-specific binding was inhibited by incubation with porcine normal serum (Dako) 1:5 in Tris-buffered saline (TBS). Each primary antibody (1:50–1:100) was applied overnight at 4 °C. The secondary antibodies (1:200) were incubated at room temperature for at least 2 h. Visualization was achieved with aminoethylcarbazole (AEC) for at least 5 min. Red staining within the tissue indicates a positive antibody reaction. After counterstaining with hematoxylin the sections were coverslipped in Aquatex (Boehringer, Mannheim, Germany). Two negative control sections were used in each case: one was incubated with the secondary antibody only, and the other one with the primary antibody only.

### Enzyme-Linked Immunosorbent Assay (ELISA)

Tear fluid of healthy donors and patients suffering from DED as well as ocular tissue samples were analyzed by quantitative sandwich ELISA. Analysis was performed by using the Human Putative Protein SFTA3 (SFTA3) ELISA kit and the regarding protocols from Cusabio Biotech Co., Ltd. (Wuhan, China). Quantification was accomplished by comparison with the 2-fold standard dilution series and the determined values for antigen concentration ranging from 15 ng/ml to 0 ng/ml. Subsequently, each sample was approximated to ng/mg.

### Interfacial tension

The method itself is described by www.kruss.de. It is an optical tensiometric method that measures the geometry of a rotating drop in the heavy phase. When a heavy phase and a light phase are situated in a horizontal, rotating capillary, the drop radius perpendicular to the axis of rotation depends on the interfacial tension γ between the phases, the angular frequency ω of the rotation and the density difference Δρ. In the classic approach according to Vonnegut’s equation, the interfacial tension can be calculated from the measured drop diameter d (=2r) with a given speed of rotation and with known densities of the two phases (www.kruss-scientific.com). Adding surface active substances changes the geometry and stability of the drop, thus enabling determination of the density-dependent influence of added proteins on the surface tension. A spinning drop interfacial tensiometer (model Kruss, Germany) was used to measure the surface and interfacial tensions of the tear film containing various concentrations of SFTA3 at room temperature.

### Wound healing assay (Scratch Assay)

Wound healing assay was performed as previously described by Schicht *et al*.^12^.

HCE cells were grown in the above-mentioned medium until confluence was reached. Using pipette tips, the cell layer was scratched several times, creating “wounds” of similar width. The cells were washed twice with PBS to remove debris, and fresh medium was applied. Images of wounded areas were taken and areas were marked for later observation. Cells were subsequently stimulated with 300 µg/ml rhu-SFTA3 (n = 4). The negative control (n = 4) contained no rhu-SFTA3 while BSA (300 µg/ml) (n = 3) served as a protein control. The previously imaged areas were photographed again after 24 hours of stimulation. The wounded area was assessed at 0 hours as well as 24 hours using Adobe Photoshop. Stimulated samples were compared to the controls.

### Statistical analysis

The statistical analysis (significance) was evaluated by using an unpaired, a two-sided Mann-Whitney *U*-test (nonparametric data) or two-sided Welch’s *t*-test (parametric data). Significance was defined at *P < 0.05, **P < 0.01 or ***P < 0.001. All data are presented as mean ± standard error of the mean (SEM).

## Results

### Expression of SFTA3 in tissues of the ocular surface, lacrimal gland and eyelids

**Figure 1:**
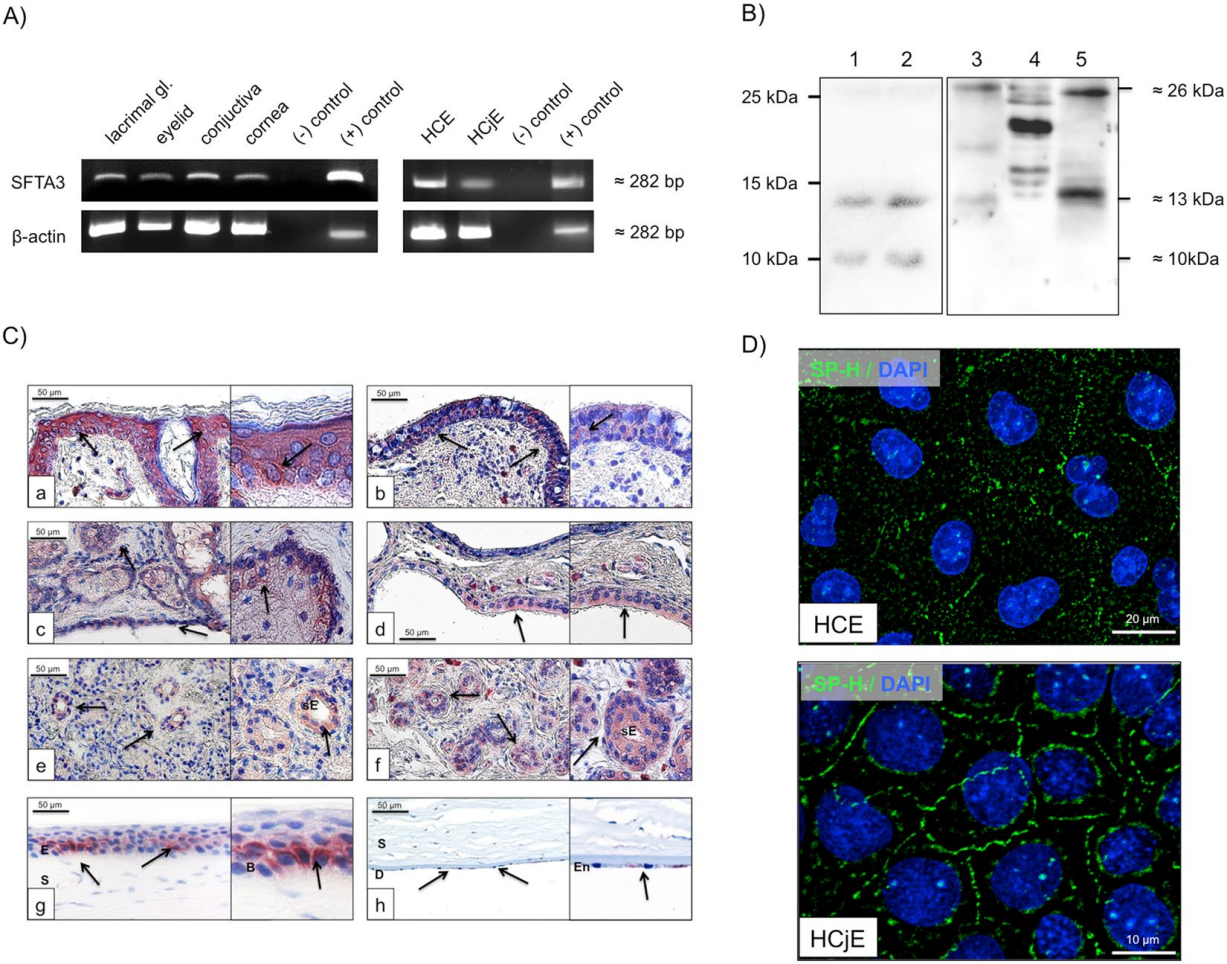
Visualization of SFTA3. (**A**) Specific detection of SFTA3 mRNA in the following samples: lacrimal gland, eyelid, conjunctiva, cornea, human corneal epithel cells (HCE), human conjunctival epithel cells (HCjE). Samples without cDNA were used as negative controls, whereas samples containing lung cDNA were used as positive controls. (**B**) Western blot analysis with a polyclonal anti-rabbit SFTA3 antibody incubated on protein extracts derived from tear film [1], tear film [2], HCE [3], HCjE [4] and HMGEC [5] (**C**) Immunohistochemical detection (red staining, black arrows) of SFTA3 in tissues of the ocular surface, lacrimal glands and eyelids. [a] Eyelid: detection in the basal epithelium of the eyelid. [b] Conjunctiva: multilayered epithelium of the conjunctiva shows weak reactivity. [c,d] Meibomian glands and Glands of Moll: SFTA3 detected within the cytoplasm of acinar cells and cells of the excretory duct system. [e,f] Lacrimal gland: detected in secretory ducts. [g] Cornea: protein detected in the basal epithelium. (s = stroma, e = epithelium, b = basal) [h] Cornea endothelium: positive staining in the endothelial cells. (**D**) HCE and HCjE cell lines: detection of SFTA3 within the plasma membrane (SFTA3: green; DAPI (4′,6-diamidino-2-phenylindole): blue).

SFTA3 mRNA was detected in tissues of the ocular surface and lacrimal duct system by RT-PCR. Tissues of lung (control), eyelids, lacrimal gland, conjunctiva and cornea were positive for SFTA3 mRNA (Fig. 1A). The loading control β-actin was used for all samples. The sequenced PCR gel electrophoresis showed bands corresponding to the expected SFTA3 DNA sequence taken from the gene bank (www.ncbi.com).

Human tear film samples were tested for presence of the SFTA3 protein using Western blot analysis (Fig. 1B). The antibodies detected distinct and SFTA3-specific protein bands at 10, 13 and 26 kDa.

### Distribution of SFTA3 within tissues of the ocular surface, lacrimal gland and eyelids

SFTA3 showed a distinct distribution pattern in tissue of the ocular system. In all investigated tissue samples antibody reactivity against SFTA3 was discovered (Fig 1C). 3–5 μm sections of paraffin-embedded tissue samples of the ocular system (eyelid, conjunctiva, lacrimal gland, cornea) were analyzed. The negative controls (primary and secondary antibody only) of every sample were always negative (i.e. unstained). The figure insets show close-up views of the used tissue. In every tissue section, a positive SFTA3 antibody reactivity is represented by red staining. Epithelium of the eyelid: SFTA3 was present in the epithelium of the eyelid. It was almost equally distributed within all layers of the epidermis (Fig. 1C-a). The subcutaneous tissue (lamina propria) showed no antibody reactivity. However, there were single subepithelial cells that stained positive for SFTA3 within their cytoplasm. These cells had the appearance of subepithelial macrophages and were only observed in some areas within the loose connective tissue of the eyelid (see also Fig. 1C-b,d). Conjunctiva: Epithelial cells of the conjunctiva showed a marked reaction with the SFTA3 antibody within the cytoplasm with the exception of the stored secretion products of intraepithelial goblet cells that did not react (Fig. 1C-b). Meibomian glands: Meibocytes were SFTA3-positive, as were the lining cells of the excretory duct system. Especially basal and differentiating meibocytes revealed pronounced reactivity whereas mature and hypermature meibocytes only showed weak staining (Fig. 1C-c,d). Glands of Moll: Cytoplasmic staining for SFTA3 was demonstrated in the epithelium of the glands of Moll. Lacrimal gland: Cells of the tubular system and to a lesser extent also the acinar cells displayed SFTA3 antibody staining (Fig. 1C-e,f). Again, single cells within the loose connective tissue between the lacrimal acini that had the appearance of macrophages showed a positive SFTA3 antibody reactivity within the cytoplasmic compartment.

Cornea: SFTA3 reactivity was present in the cytoplasm of basal epithelial cells (Fig. 1C-g). Also endothelial cells showed a weak reactivity with the SFTA3 antibody (Fig. 1C-h), whereas stromal cells did not react at all with the SFTA3 antibody. The immunocytochemical investigations on corneal (HCE) and conjunctival epithelia celllines (HCjE) showed that SFTA3 is localized on the outer cell membrane and within the cytoplasm (Fig. 1D).

### Quantification of SFTA3 in tissues of the ocular surface, lacrimal gland and eyelids

**Figure 2:**
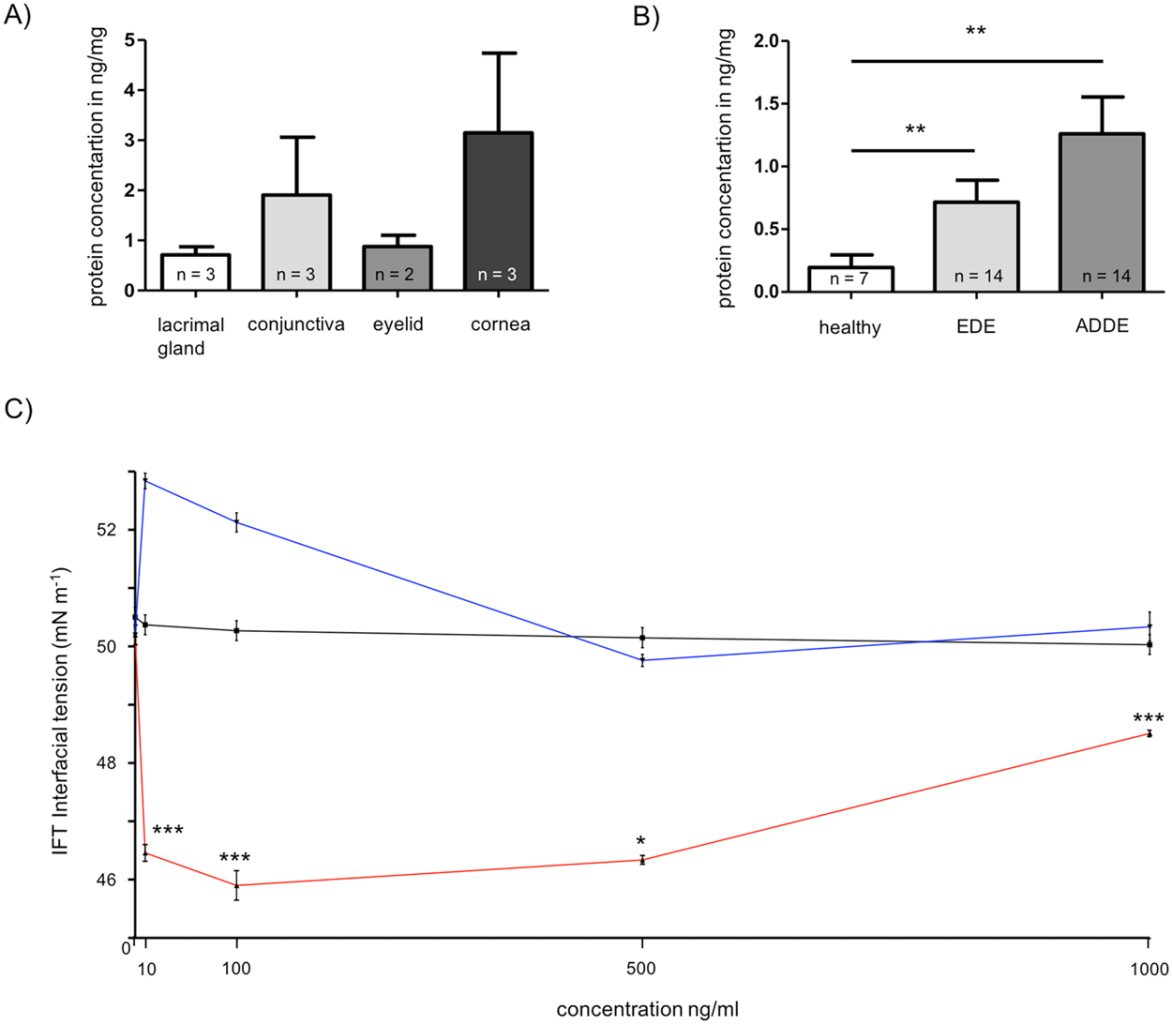
Protein quantification of SFTA3 in tissues of the ocular surface as well as tear film and interfacial tension analysis. (**A**) Quantification of SFTA3 in ocular tissue samples. Mean values of lacrimal gland: 0.712 ng/mg, conjunctiva: 1.908 ng/mg, eyelid: 0.875 ng/mg and in cornea 3.14 ng/mg. (**B**) Quantification of SFTA3 in tear film. Mean values of healthy subjects: 0.196 ng/mg, EDE: 0.669 ng/mg and in ADDE: 1.157 ng/mg. The protein concentration is expressed in ng/mg and as mean ± SEM. Statistical significance: *P ≤ 0.05, **P ≤ 0.005, ***P ≤ 0.0005. (**C**) Interfacial tension (IFT) for different rhu-SFTA3 concentrations in tear film samples (red). As control we used water (black) and BSA (blue) as protein loading control. The IFT is expressed in mN/m^−1^ and as mean ± SEM. Statistical significance: *P ≤ 0.05, **P ≤ 0.005, ***P ≤ 0.0005.

Comparative quantification of the SFTA3 content in tear fluid of the two DED forms yielded an average value of 1.157 ng/mg (n = 14) in samples of patients with aqueous deficient dry eye (ADDE, i.e. lack of tears) and of 0.669 ng/mg (n = 14) in patients suffering from evaporative dry eye (EDE). Healthy donors exhibited an average SFTA3 protein concentration of 0.196 ng/mg (n = 7) (Fig. 2B). Hence, the content of SFTA3 protein within the tear fluid showed a significant increase in both cases of DED (i.e. ADDE and EDE) compared to healthy subjects (Fig. 2B). The mean SFTA3 concentrations in ocular tissue samples as quantified by ELISA were 0.875 ng/mg in the eyelid (n = 2), 0.712 ng/mg in the lacrimal gland (n = 3), 1.908 ng/mg in the conjunctiva (n = 3) and 3.14 ng/mg the in cornea (n = 3) (Fig. 2A).

### SFTA3 activity in tear film

Surfactant proteins are characterized by a high inherent surface activity and a certain level of hydrophobic surface exposure. The presence of SFAT3 within the tear film resulted in a significant decrease in water surface tension (IFT) from 50 to 46 mN/m^−1^ as determined by Spinning Drop Method (n = 3). BSA as loading protein control shows no effects (Fig. 2C).

### Scratch assay data

**Figure 3:**
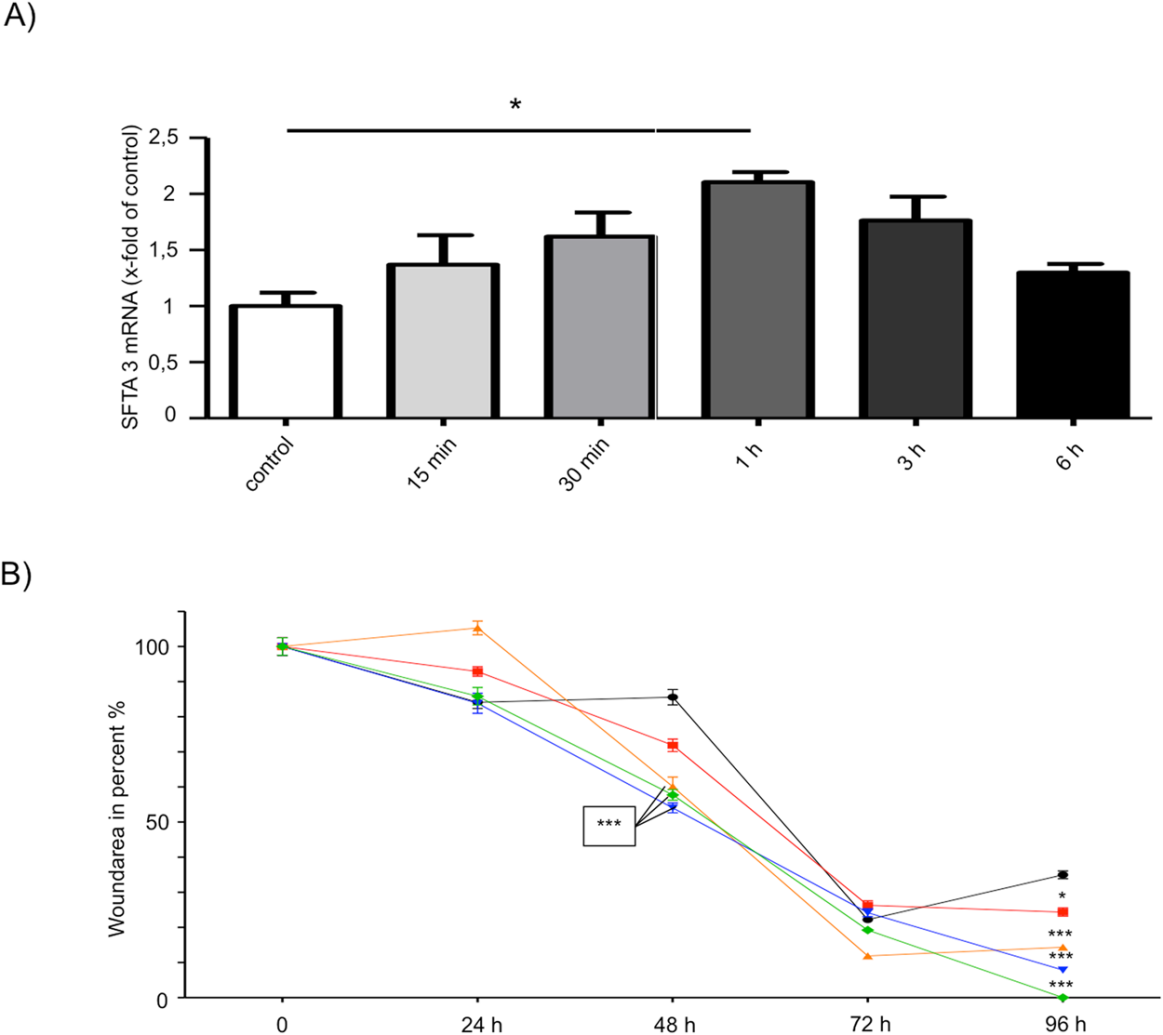
*In vitro* wound healing scratch assay with HCE cells (**A)** Scratching of a HCE cell monolayer and quantification of SFTA3 mRNA by Real Time PCR after a wound healing period of 15 min; 30 min; 1; 3 and 6 hours, respectively. Statistical significance of the difference to the controls after 1 h only (n = 3; *P ≤ 0.05). The regulation of SFTA3 transcript levels was expressed as mean ± SEM. (**B**) Scratch assay on HCE cells. Percentages of wounded area in HCE monolayer cultures incubated with 5 (red), 10 (yellow), 100 (blue), 500 (green) µg/ml rhu-SFTA3 were compared to control (black) values (no treatment). The wound healing rates (closure of the scratch) were significantly higher under stimulation with 500 µg/ml rhu-SFTA3 compared with the control (no rhu-SFTA3). Wounded areas were reduced significantly after 48 and 96 hours of incubation. The wound healing area was expressed as mean ± SEM. Statistical significance: *P ≤ 0.05, **P ≤ 0.005, ***P ≤ 0.0005.

Corneal epithelial cell damage increases SFTA3 mRNA expression *in vitro*. The corneal epithelial cell line (HCE) expresses SFTA3 and scratching of HCE cells cultured as a monolayer leads to a significant increase in SFTA3 expression. Real Time RT-PCR analysis showed a significant increase in SFTA3 mRNA expression within the first hour after scratching (Fig. 3A, Table 1). After 3 hours the SFTA3 mRNA expression had markedly decreased and after 6 hours it had returned back to the basal level.

**Table 1:** Real-time RT-PCR (corneal epithelial cell line (HCE)): upregulations of SFTA3 expression compared to control, which was normalized to 1.

	0 min	15 min	30 min	1 h	3 h	6 h
SFTA3 Ø	0,8482	1,161	1,373	1,786	1,496	1,102
SEM (±)	0,1396	0,2979	0,2409	0,104	0,3018	0,0998

Data’s are presented as mean (Ø) with standard error of the mean deviation (SEM/±).

SFTA3 increased the wound closure rate *in vitro*. The cell culture-based wound healing assay (n = 4) revealed an increased gap closure by HCE cells in the presence of 5, 10, 100 and 500 μg/ml SFTA3 compared to 0 µg/ml (Fig. 3B, Table 2). After incubation with SFTA3 for 12, 24, 48, 72, and 96 hours, the remaining wound area was significantly smaller when compared to the controls. The most pronounced wound closure rate was observed after a 96-hour incubation with 500 μg/ml of SFTA3.

**Table 2:** Wound healing assay (corneal epithelial cell line (HCE)): Wounded area in percent (%), data’s are presented as mean (Ø) with standard error of the mean deviation (SEM/±).

	0 µg/ml	5 µg/ml	10 µg/ml	100 µg/ml	500 µg/ml
0 h	Ø 100% (±2,48)	Ø 100% (±2,48)	Ø 100% (±2,48)	Ø 100% (±2,48)	Ø 100% (±2,48)
24 h	Ø 84,13% (±1,74)	Ø 92,9% (±1,35)	Ø 105,3% (±1,96)	Ø 83,85% (±2,85)	Ø 85,85% (±2,50)
48 h	Ø 85,58% (±2,19)	Ø 71,87% (±1,78)	Ø 60,12% (±2,71)	Ø 54,05% (±1,40)	Ø 57,76% (±1,52)
72 h	Ø 22,21% (±0,87)	Ø 26,34% (±1,26)	Ø 11,88% (±0,65)	Ø 24,21% (±1,13)	Ø 19,23% (±0,81)
96 h	Ø 34,98% (±1,10)	Ø 24,36% (±1,18)	Ø 14,35% (±0,93)	Ø 7,846% (±0,55)	Ø 0% (±0)

### Effects of bacterial supernatants on SFTA3 expression

**Figure 4:**
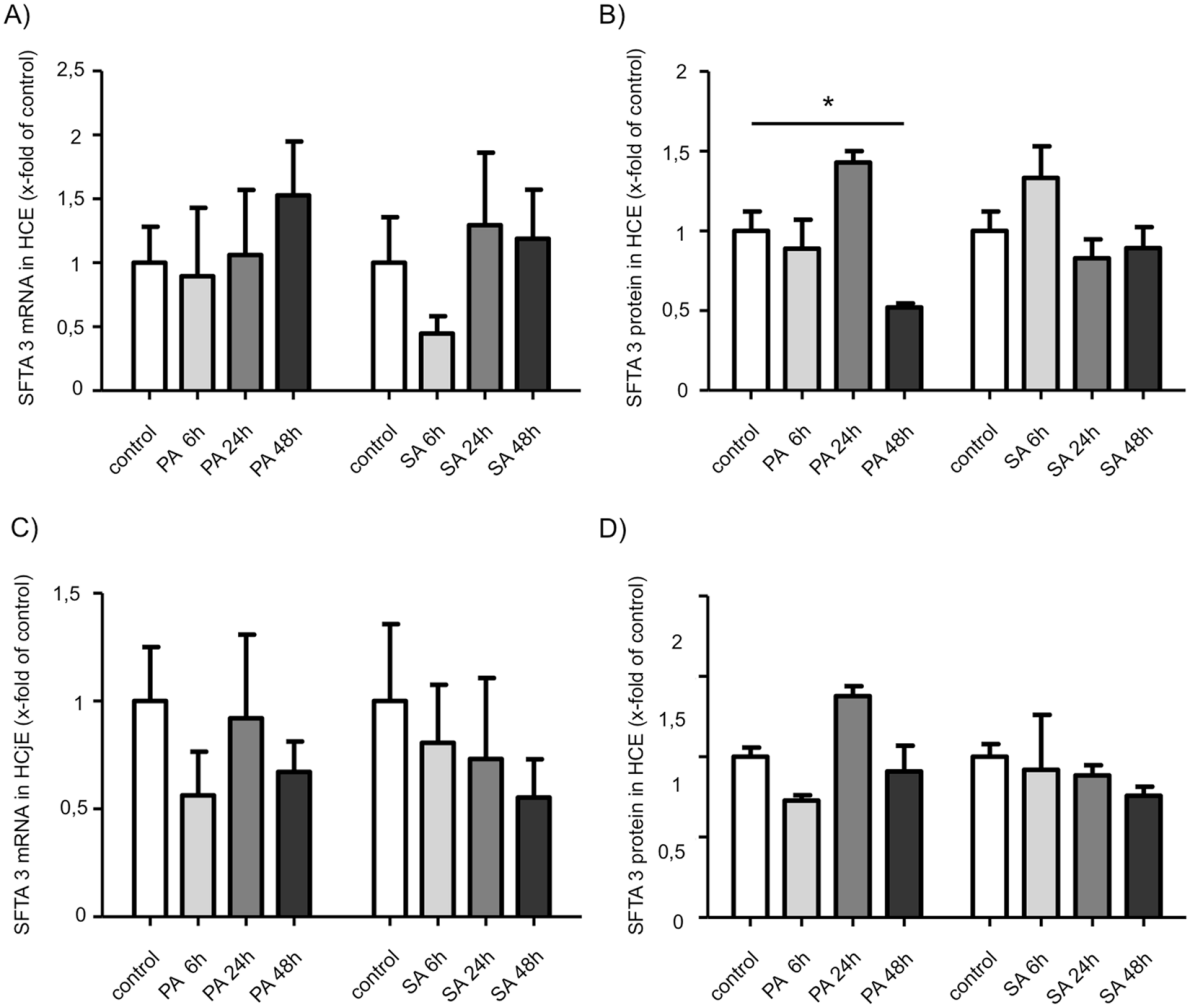
SFTA3 expression after stimulation with bacterial supernatant. Stimulation of HCE cells **(A**,**B**) for 6; 24 and 48 hours with a 1:100 dilution of bacterial supernatant of *Pseudomonas aeruginosa* (PA) and *Staphylococcus aureus* (SA). (**A**) Relative quantification of SFTA3 mRNA by Real Time PCR. No statistical significance of the differences after 6; 24 and 48 hours (n = 3). (**B**) Relative quantification of SFTA3 protein by ELISA shows a statistically significant decrease after 48 hours for PA (n = 3; *P ≤ 0.05). No statistical significance after 6; 24 and 48 hours (n = 3) for SA. (**C)** Relative quantification of SFTA3 mRNA by Real Time PCR shows no statistical significance after 6; 24 and 48 hours (n = 3) for SA and PA. (**D**) Relative quantification of SFTA3 protein by ELISA shows no statistical significance after 6; 24 and 48 hours (n = 3) for SA and PA.

**Figure 5:**
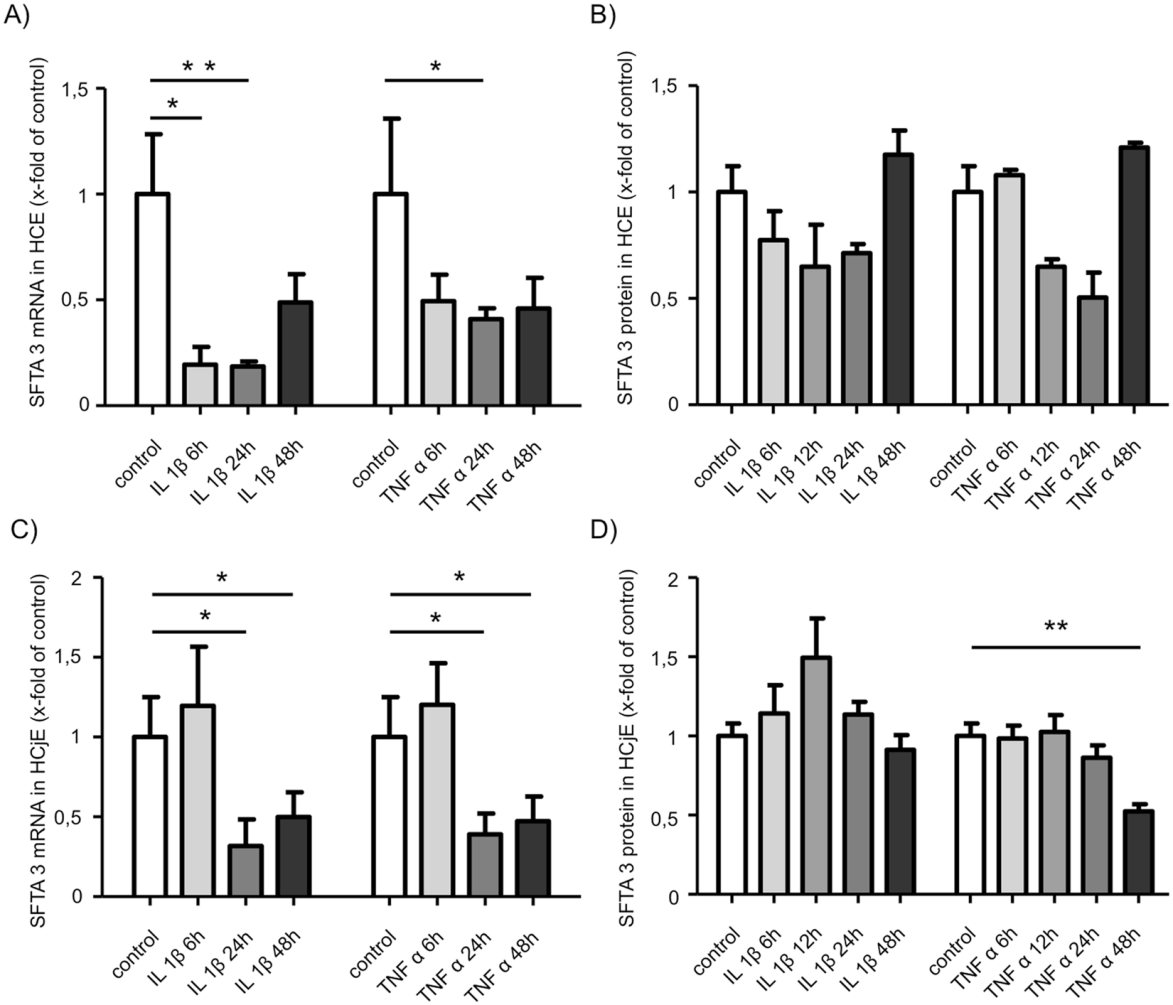
Influence of cytokines on the expression of SFTA3 in the cell lines HCE and HCjE. Stimulation of HCE cells **(A**,**B)** for 6; 12; 24 and 48 hours with 10 ng/ml of recombinant IL-1β or TNFα, respectively. Statistical significance of the decrease in SFTA3 mRNA expression after 6 and 12 hours for IL-1β (n = 3; *P ≤ 0.05, **P ≤ 0.005) and after 24 hours for TNFα (n = 3; *P ≤ 0.05). (**A**) Relative quantification of SFTA3 mRNA by Real Time PCR. (**B**) Relative quantification of SFTA3 protein by ELISA showed no statistically significant differences. Stimulation of HCjE cells **(C**,**D)** for 6; 12; 24 and 48 hours with 10 ng/ml of recombinant IL-1β or TNFα, respectively. Statistically significant decrease in SFTA3 mRNA expression after 24 and 48 hours for IL-1β and TNFα (n = 3; *P ≤ 0.05). (**C**) Relative quantification of SFTA3 mRNA by Real Time PCR. (**D**) Relative quantification of SFTA3 protein by ELISA. Statistically significant reduction of SFTA3 protein after 48 hours for TNFα (n = 3; *P ≤ 0.05).

Stimulation experiments with inflammatory cytokines and supernatants of PA and SA showed no negative effects on SFTA3 expression in cultivated HCE and HCjE cells. No significant regulation of SFTA3 mRNA concentration was detected after stimulation with supernatants of PA and SA (Fig. 4A,C). On protein level SFTA3 was significantly diminished after 48 h incubation of HCE cells with PA supernatant as shown by ELISA (Fig. 4B). No significant alterations on protein level were observed in HCjE cells (Fig. 4C,D). By contrast, the stimulation of both cell lines with interleukin−1beta (IL-1β) and TNFα showed a significant down-regulation of SFTA3 mRNA within 24 h *in vitro* (Fig. 5A,C). Also, data obtained from ELISA experiments revealed a significant decrease of SFTA3 protein expression after 48 h incubation of HCjE cells with TNFα (Fig. 5D). A slight but not significant decrease was also observed in HCE cells stimulated for at least 48 h with IL-1β or TNFα and in HCjE cells stimulated with IL-1β (Fig. 5B).

## Discussion

The SFTA3 gene was first demonstrated in developing lung tissue, bronchoalveolar lavage (BAL) and the immortalized alveolar type II cell line A549^12^. Here, we demonstrate that SFTA3 is a component of the lacrimal apparatus. It is produced by different tissues of the ocular system like cornea, conjuctiva, lacrimal gland, eyelid and efferent tear ducts. From these tissues, SFTA3 is secreted into the tear film.

Western blot analysis of tear fluid revealed three distinct protein bands at 10, 13 and 26 kDa, respectively. This SFTA3 expresion pattern is in accordance with findings in lung tissue, bronchoalveolar lavage (BAL) and the immortalized alveolar type II cell line A549^12^. Further data obtained from our ELISA experiments disclosed that cells of the eyelid and the lacrimal gland show a lower concentration of SFTA3 compared to cornea and conjunctiva.

The results clearly show that the two established cell lines HCE and HCjE produce and secrete SFTA3 and can be used for further stimulation experiments. The cell lines are useful as an *“in vitro”* ocular surface model for further SFTA3 studies. SFTA3 was detected in acinar cells of the lacrimal gland, as well as in conjunctival epithelial cells and efferent tear duct epithelial cells. Goblet cells were always SFTA3-negative. In addition, SFTA3 was shown to be secreted into the tear film. This corresponds to previous reports on SFTA3 being detected in bronchial epithelium and alveolar parenchyma. As far as the cornea is concerned, it is important to point out that SFTA3 was detected in the basal epithelial cells and in the endothelial cells, only. This indicates that the SFTA3 produced by the basal epithelial cells is not secreted into the tear film, but rather serves a different function. Which consequences this may have for organisms, must be clarified by further research. We were able to show that SFTA3 is synthesized by meibocytes and the epithelial cells of the excretory ducts of the meibomian glands. It can therefore be assumed that SFTA3 is a secretory product of the meibomian glands and interacts with the lipids to facilitate the secretion process. This is in line with previous reports on meibocytes producing different proteins that interact with the lipids of meibum^27^.

Interestingly, we found that the SFTA3-protein concentration is increased in tears from patients suffering from different forms of DED. We suppose that there are several possible explanations for this. For example, SFTA3 expression may be increased in order to counter balance the increased evaporation in the case of EDE in which the lipid layer is diminished. As shown by immunofluorescence, SFTA3 is localized in the plasma membrane of HCE and HCjE cells, indicating its ability to interact with hydrophobic groups and therefore may possess surface active properties. We believe that SFTA3 can be considered an additional regulatory factor to influence surface tension. This assumption is supported by previous molecular dynamic (MD) simulations, which showed a theoretical interaction potential for SFTA3 with lipid systems^12^.

Beside its function to regulate the relative loss of tear fluid, another explanation for the DED-related increase of SFTA3 in the tear fluid may be a potential role in humoral immunity. As it is known that DED is accompanied by inflammation and sometimes bacterial infection, these pathologic states may cause an increased expression of SFTA3. A general increase in the expression of all surfactant proteins (SP-A, SP-B, SP-C and SP-D) in patients suffering from DED has been described earlier^28^. However, to date, it remains unclear whether this increase is the cause or an effect of DED. The observed increased expression of surfactant proteins could, for example, result in a destabilization of the tear film. On the other hand, it possibly gives rise to several other proinflammatory mechanisms (e.g. release of reactive oxygen species, increased expression of adhesion molecules, modulation of T-lymphocytes and dendritic cells)^29,30^ that further exacerbate the severity of DED symptoms. It is as yet unclear, if the observed higher SFTA3 concentration in the tear film of DED patients is beneficial for the stabilization of the tear film.

The spinning drop analysis showed that additional recombinant SFTA3 reduces the surface tension in tears. This indicates the high potential of SFTA3 to interact with a membrane system and supports our hypothesis that SFTA3 is able to interact with lipid systems. Therefore, it is tempting to speculate that high concentrations of SFTA3, in the tear film of DED patients reduce the surface tension and destabilize the tear film.

Beside its yet unknown involvement in the context of DED, our *in vitro* data suggest a potential role of SFTA3 in the context of post-injury/healing processes in the human cornea. Accordingly, we found a short term regulation of SFTA3 after epithelial damage as demonstrated by quantitative Real Time PCR after *in vitro* scratch assay. SFTA3 expression reached its maximum one hour after induction of epithelial damage. Then, it declined and gradually returned to its basal level 6 hours after the scratch. Incubation with rhu-SFTA3 resulted in a significant reduction of the wound area after 96 h. Another peptide that was recently reported to be involved in epithelial healing in the context of dry eye disease is trefoil factor family peptide 3 (TFF3). Compared with TFF3, which remains on its basal expression level within the first hour after the scratch and reaches its most obvious change in expression six hours after setting of the lesion^31^, SFTA3 displays a quite early and temporally limited reaction to epithelial damage. Our results confirmed the assumption that SFTA3 might be involved in the initiation of repair mechanisms within the corneal epithelium, maybe contributing to the release of other wound healing factors like EGF, FGF or TFF3. Because of the importance of this finding additional experiments using a mouse model for corneal damage/wound healing are crucial in order to determine the exact relevance and function of SFTA3.

To reveal the influence of bacterial infection and proinflammatory cytokines on SFTA3 expression in ocular tissue we performed experiments using HCE and HCjE cell lines as previously described. These cells are well established and known to produce surfactant proteins in general^5,6^. HCE- and HCjE cells were incubated with bacterial supernatants of SA and PA as both bacteria are common causes of bacterial keratitis, an inflammatory process that can lead to loss of vision^32^. Although our results revealed no considerable effect on SFTA3 expression after stimulation with bacterial supernatant of SA and PA, it cannot be excluded that the expression of SFTA3 is dependent on the immunological activation state and/or the presence of distinct bacterial strains. Therefore, the use of bacterial strains directly isolated from the ocular surface may represent a more suitable model for bacterial infections of the eye in cell culture experiments.

Recent studies have shown that the pro-inflammatory cytokines IL-1β and TNFα are elevated in DED patients^33^. Interestingly, stimulation of HCE- and HCjE cells with IL-1β or TNFα resulted in a down-regulation of SFTA3 mRNA *in vitro*. But as a significant effect on protein level could only be demonstrated for the conjunctival epithelial cell line HCjE 48 h after stimulation with TNFα, it is possible that these two cytokines are no direct modulators SFTA3 expression.

The reduction of SFTA3 expression by inflammatory components suggests that SFTA3 does not play an important role in the context of ocular immune responses.

Up to now, considerations concerning the function of SFTA3 are still highly speculative. We are convinced, however, that SFTA3 is a regulator of surface tension and has rheological potential. Combined with the wound healing effects SFTA3 seems to constitute an important factor for the homeostasis of the ocular surface.
